# Zebrafish androgen receptor is required for spermatogenesis and maintenance of ovarian function

**DOI:** 10.18632/oncotarget.24407

**Published:** 2018-02-06

**Authors:** Guangqing Yu, Dawei Zhang, Wei Liu, Jing Wang, Xing Liu, Chi Zhou, Jianfang Gui, Wuhan Xiao

**Affiliations:** ^1^ State Key Laboratory of Freshwater Ecology and Biotechnology, Institute of Hydrobiology, Chinese Academy of Sciences, Wuhan, P. R. China; ^2^ The Key Laboratory of Aquatic Biodiversity and Conservation, Institute of Hydrobiology, Chinese Academy of Sciences, Wuhan, P. R. China; ^3^ The Key Laboratory of Aquaculture Disease Control, Ministry of Agriculture, Wuhan, P. R. China; ^4^ University of Chinese Academy of Sciences, Beijing, P. R. China

**Keywords:** zebrafish, androgen receptor, spermatogenesis, ovarian function

## Abstract

The androgen receptor (AR) is a nuclear receptor protein family member and inducible transcription factor that modulates androgen target gene expression. Studies using a mouse model confirmed the need for *ar* in reproductive development, particularly spermatogenesis. Here, we investigated the role of *ar* in zebrafish using CRISPR/Cas9 gene targeting technology. Targeted disruption of *ar* in zebrafish increases the number of female offspring and increases offspring weight. In addition, *ar*-null male zebrafish have female secondary sex characteristics. More importantly, targeted disruption of *ar* in zebrafish causes male infertility via defective spermatogenesis and female premature ovarian failure during growth. Mechanistic assays suggest that these effects are caused by fewer proliferated cells and more apoptotic cells in *ar*-null testes. Moreover, genes involved in reproductive development, estradiol induction and hormone synthesis were dys-regulated in testes and ovaries and the reproductive-endocrine axis was disordered. Our data thus suggest that the zebrafish *ar* is required for spermatogenesis and maintenance of ovarian function, which confirms evolutionarily conserved functions of *ar* in vertebrates, as well as indicates that *ar*-null zebrafish are a suitable model for studying pathologic mechanisms related to androgen disorders.

## INTRODUCTION

The androgen receptor (AR) is a member of the nuclear receptor family of proteins that acts as ligand-inducible transcription factors, which comprises three main functional domains: the N-terminal transcriptional domain, the DNA binding domain (DBD) and the ligand binding domain (LBD) [1]. Testosterone and its more potent metabolite, dihydrotestosterone (DHT), can bind *ar*. Upon androgen binding, the *ar* forms a dimer, translocates to nucleus and recruits a wide variety of co-regulators to modulate androgen target genes [2]. Clinical disorders related to *ar* dysfunction include testicular feminization mutation syndrome (Tfm), prostate cancer and Kennedy's disease [3–6]. In addition, the Tfm syndrome has been observed in other mammals [7–9].

To elucidate the molecular basis of *ar*-related disorders, in the last decade, generation and characterization of *ar* knockout mouse models (ARKO) revealed key roles of the *ar* in male and female reproduction [10–12]. Because the mammalian *ar* gene is located on the X chromosome, which is critical for male fertility, it is impractical to generate an ARKO mouse line using conventional gene targeting. To date, most data about ARKO mice are obtained from a Cre-loxP strategy for conditional KO, including global and cell-specific androgen receptor KO mice [11, 13–28]. The *ar* mediates androgen actions on different parts of the reproductive system at different stages of development. In males, *ar*-mediated androgen activity is involved in differentiation of efferent duct system for germ cells [29], spermatogenesis [25], sex and reproductive behavior, and secondary sex characteristics [12, 30, 31]. In females, the *ar*-mediated androgen actions are important for reproductive development and function, including folliculogenesis, and uterine and mammary gland development [16, 32–35].

The failure of product amplification from lampreys using many combinations of degenerate *ar* primers suggests that *ar* gene may be specific to jawed vertebrates [36]. A jawed vertebrate such as teleost fish have the *ar* gene, and androgens are involved in secondary sex characteristics and behavior [37, 38], spermatogenesis [39, 40], and Leydig cell androgen production [41]. Moreover, fish appear to be more sensitive to androgen with respect to sex differentiation because fully functional female-to-male sex reversal can be induced by exposure of juvenile or adult fish to androgens [42, 43].

Zebrafish (*Danio rerio*), as one of the model organisms, their *ar* gene has been isolated and its expression patterns and biochemical characteristics have been described [44–46]. However, whether zebrafish *ar* has effects on sex determination or gonad development is still largely unknown. Molecular control of sex determination and gonad differentiation in zebrafish appears to be complex and variable across domesticated strains versus wild populations [16, 47–49]. Zebrafish belong to undifferentiated gonochoristic fishes: during the juvenile period, all individuals develop undifferentiated ovary-like gonads containing immature oocytes. Between 20 and 30 days post fertilization (d.p.f.), some immature oocytes develop into ovaries, while the other immature oocytes degenerate and acquire testis morphology [50–52].

In this study, we used CRISPR/Cas9 technology to investigate whether *ar* plays a role in reproductive development in zebrafish. Loss of *ar* caused progressive loss of spermatogenesis and ovarian function, so we suggest that the *ar* is key to spermatogenesis and maintenance of ovarian function.

## RESULTS

### Targeted disruption of *ar* produces more female zebrafish and increases weight in male and female zebrafish

**Figure 1:**
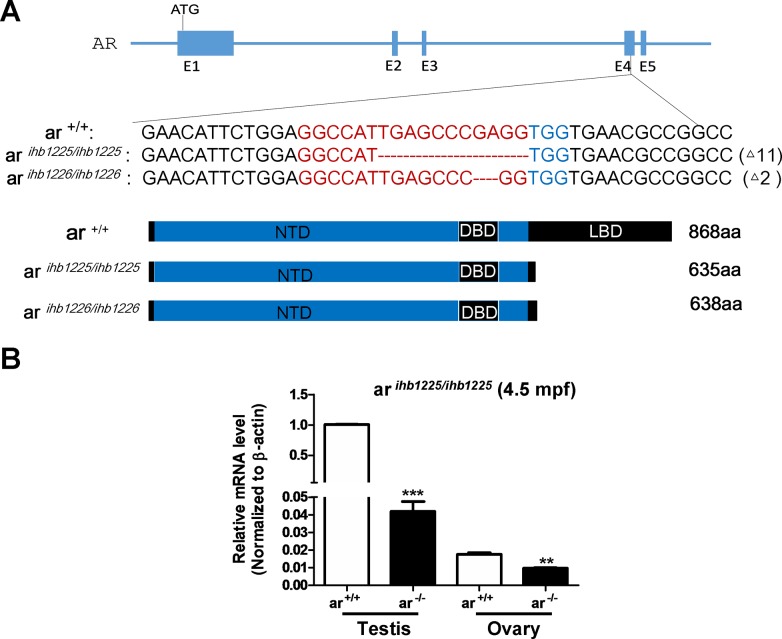
Generation of *ar*-null zebrafish via CRISPR/Cas9 technology **(A)** Scheme of the genomic structure of zebrafish *ar*, the sequence information of targeting sites and the predicted protein products of *ar* in the mutants *ar ^ihb1225/ihb1225^* and *ar ^ihb1226/ihb1226^*. gRNA binding site sequences are highlighted in red font. *ar* ^+/+^, wild-type; *ar ^ihb1225/ihb1225^*, homozygous mutant line 1 (*ar* ^-/-^) with 11-bp nucleotides (TGAGCCCGAGG) deletion in the Exon 4 of *ar*; *ar ^ihb1226/ihb1226^*, homozygous mutant line 2 (*ar* ^-/-^) with 2-bp nucleotides (GA) deletion in the Exon 4 of *ar*. **(B)** Expression of *ar* in testes or ovaries from the wildtype (*ar* ^+/+^) and the *ar*-null mutant 1 (*ar*^-/-^) zebrafish (4.5 mpf; n=3, respectively). Mpf, month post fertilization.

*ar* is evolutionarily conserved among zebrafish, mouse, rat and humans, particularly in its DNA-binding domain (DBD) and ligand-binding domain (LBD) (Supplementary Figure 1). After micro-injecting synthesized sgRNA and Cas9 mRNA into one-cell stage embryos, we initially used HMA (Heteroduplex mobility assay) to measure the efficiency of sgRNA/Cas9-mediated *ar* disruption in the F0 generation and then conducted sequencing confirmation of the F1 generation. After screening, we obtained two mutants in the *ar* gene (*ar ^ihb1225/ihb1225^* and *ar ^ihb1226/ihb1226^*, respectively; Figure 1A and Supplementary Figure 2). In these two mutants, two truncated peptides were predicted which lacked most of the LBD (Figure 1A). Reduced *ar* mRNA in testes and ovaries was confirmed in *ar ^ihb1225/ihb1225^* adult *ar*-null zebrafish (Figure 1B), which might be due to non-sense mediated decay.

**Figure 2:**
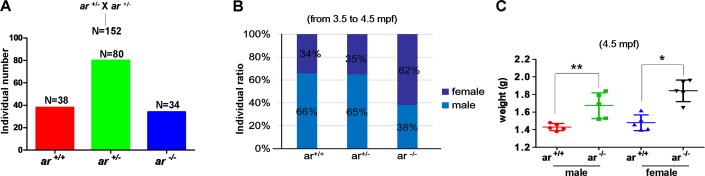
Targeted disruption of *ar* produces more female zebrafish and increases weight increased in male and female zebrafish **(A)** The individual number of three genotypes from the offspring of intercrossing between *ar* heterozygous (*ar* ^+/-^). **(B)** Comparison of sex ratio among wildtype (*ar* ^+/+^), heterozygous (*ar* ^+/-^) and homozygous (*ar* ^-/-^) (M1)zebrafish similarly raised. Counts were from 3.5 to 4.5 mpf zebrafish. **(C)** Weights compared between wildtype (*ar* ^+/+^) and homozygous (*ar* ^-/-^; M1) male and female zebrafish at 4.5 mpf. Mpf, month post fertilization.

From the embryonic stage to adulthood, *ar* heterozygotes (*ar*^+/−^) were indistinguishable from wildtype siblings (*ar*^+/+^) with *ar ^ihb1225/ihb1225^* or *ar ^ihb1226/ihb1226^* background. To obtain *ar* homozygotes (*ar*^−/−^), we mated *ar*^+/−^ × *ar*^+/−^. For offspring of *ar*^+/−^ × *ar*^+/−^, numbers of *ar*^+/+^, *ar*^+/−^ and *ar*^−/−^ were determined by Mendel's ratio (1:2:1) (Figure 2A), which suggested that knockout of *ar* in zebrafish had no effect on the survival. However, at the adult stage (from 3.5 mpf to 4.5 mpf), female ratio of *ar* ^−/−^ was higher than that of *ar* ^+/+^ or *ar*^+/−^ (Figure 2B). Notably, male and female *ar*^−/−^ weights were greater than wildtype siblings (*ar*^+/+^) (Figure 2C). Dissection revealed more fatty abdomens in *ar*^−/−^ males (Supplementary Figure 3). Therefore, targeted disruption of *ar* produces more female zebrafish and increases their weight.

### Ar-null male zebrafish display female secondary sex characteristics

**Figure 3:**
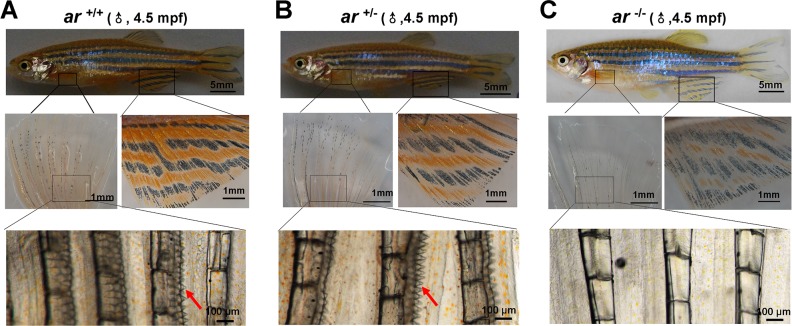
*ar*-null male zebrafish have female secondary sex characteristics **(A-C)** Wildtype (*ar*^+/+^) and heterozygous (*ar*^+/-^) male adult zebrafish have deep yellow pigmentation of the anal fin and breeding tubercles (BT) in the pectoral fin (red arrows); *ar*-null male zebrafish (*ar* ^-/-^; *ar ^ihb1225/ihb1225^*) had light yellow pigmentation of the anal fin and no pectoral BTs; *ar*-null male zebrafish (*ar*^-/-^; *ar ^ihb1225/ihb1225^*) also had a female-like appearance and a larger abdomen. Mpf, month post fertilization.

Adult wildtype and heterozygous males (*ar* ^+/+^ and *ar* ^+/−^) had deep yellow pigmentation of the anal fin and breeding tubercles (BT) (or epidermal tubercles, ET) on pectoral fins (red arrows) (Figure 3A and 3B). However, *ar*-null males (*ar* ^−/−^)(*ar ^ihb1225/ihb1225^*) had light yellow pigmentation of the anal fin and no BTs of the pectoral fin (Figure 3C). In addition, *ar*-null male zebrafish (*ar* ^−/−^)(*ar ^ihb1225/ihb1225^*) had a female-like appearance with larger abdomens (Figure 3C). Mating activity of *ar* ^−/−^ male and wildtype females were not observed so we speculate this to mean that *ar*-null male zebrafish had female secondary sex characteristics and a female-like appearance.

### Targeted disruption of *ar* in zebrafish causes male infertility with defective spermatogenesis

**Figure 4:**
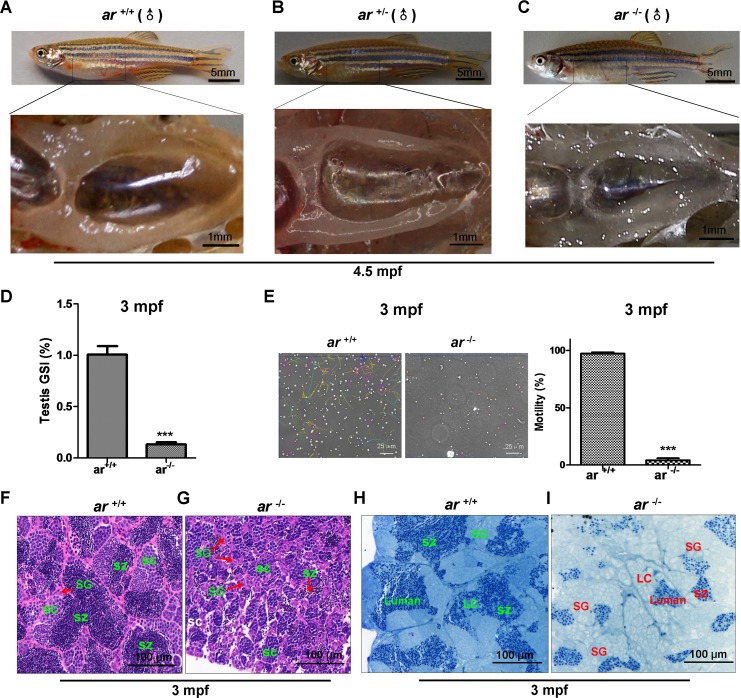
Targeted disruption of *ar* in zebrafish causes male infertility due to defective spermatogenesis **(A-C)** Gross appearance of testes from wildtype (*ar* ^+/+^), heterozygous (*ar* ^+/-^) and homozygous (*ar* ^-/-^) (*ar ^ihb1225/ihb1225^*) zebrafish. Testes of *ar* ^-/-^ male zebrafish was smaller and more transparent compared with that in their *ar* ^+/+^ or *ar* ^+/-^ male siblings. **(D)** Gonadosomatic index (GSI) in *ar*^+/+^ and *ar*^-/-^ male zebrafish. **(E)** Sperm motility evaluation under a dark-phase microscope. **(F, G)** Histology of testes from wildtype (*ar* ^+/+^) and homozygous (*ar* ^-/-^) (*ar ^ihb1225/ihb1225^*) zebrafish at 3 mpf. Compared with wildtype sibling testes, spermatogenesis in *ar* ^-/-^ testes was delayed as indicated by more spermatogonia (SG), fewer spermatocytes (SC) and fewer spermatozoa (SZ); the spermatogenetic cysts were smaller; and spermatogenesis was arrested at the second meiosis. **(H, I)** Toluidine blue staining testes from wildtype (*ar* ^+/+^) and homozygous (*ar* ^-/-^) (*ar ^ihb1225/ihb1225^*) zebrafish at 3 mpf. Compared with wildtype sibling testes, the size of spermatogonia (SG) was bigger and the number of Sertoli cells was increased in *ar* ^-/-^ testes. Leydig cells (LC) and lumen are indicated. Dpf, days post fertilization; Mpf, months post fertilization.

To understand the effect of *ar* on male reproductive development, we immunohistochemically and histologically studied testes. Grossly, testes in *ar*^−/−^ adult males were smaller and more transparent compared to *ar* ^+/+^ or *ar* ^+/−^ male siblings (Figure 4A-4C). Notably, a significant decrease of the gonadosomatic index (GSI) was found in *ar*^−/−^ testes (Figure 4D). Sperm motility assessment showed that the motility of sperms isolated from *ar* ^−/−^ testes was much lower than that of sperms isolated from *ar* ^+/+^ testes (Figure 4E). At 1.5 mpf (months post fertilization), no obvious difference were noted between *ar* ^−/−^ and their wildtype sibling's (*ar* ^+/+^) testes (Supplementary Figure 5A and 5B). However, at 70 dpf (days post fertilization), compared with the wildtype sibling testes, spermatogenesis in *ar* ^−/−^ testes was delayed and had more spermatogonia (SG) and fewer spermatocytes (SC), but no spermatozoa (SZ)(Supplementary Figure 5C and 5D). Apparently, spermatogenesis in *ar*^−/−^ testes was arrested at the first meiosis. At 3 mpf, spermatogenetic cysts were smaller in *ar* ^−/−^ testes, the proportion of SC was increased, but fewer SZ. Spermatogenesis was arrested at the second meiosis (Figure 4F and 4G). In addition, the size of spermatogonia (SG) was bigger and the number of Sertoli cells was increased in *ar* ^−/−^ testes (Figure 4H and 4I). At 6 mpf, *ar*^−/−^ testes degenerated and were loose (Supplementary Figure 5E and 5F).

**Figure 5:**
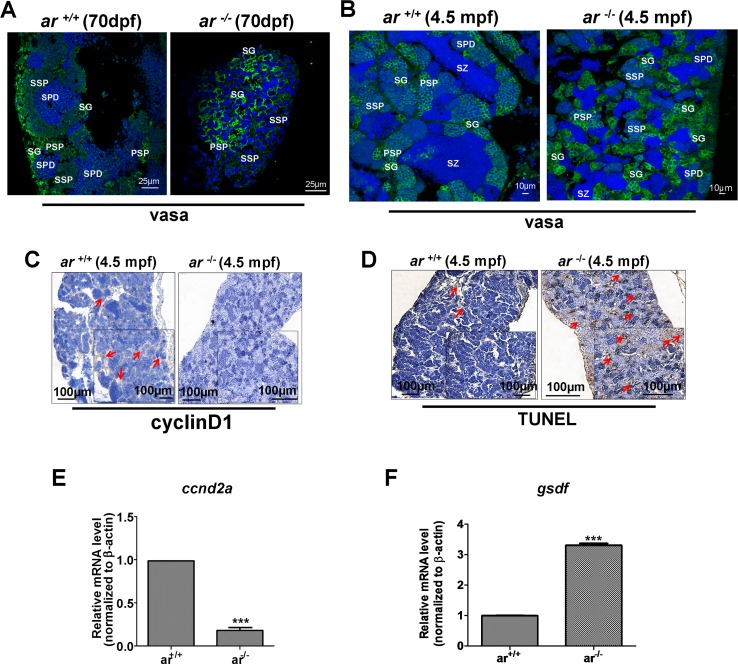
Proliferating germ cells were reduced, but apoptotic germ cells increased in *ar*-null zebrafish testes **(A, B)** Immunofluorescent staining with anti-vasa antibody identified different types of spermatogenic cells in *ar* ^-/-^ testes and wildtype sibling (*ar* ^+/+^) testes at 70 dpf or 4.5 mpf, respectively. SG, spermatogonia; SZ, spermatozoa; PSP, primary spermatocyte; SSP, secondary spermatocyte; SPD, spermatid. **(C)** Immunohistochemistry with anti-cyclin D1 antibody indicated that proliferating germ cells were reduced in *ar* ^-/-^ testes compared with wildtype (*ar*^+/+^) siblings at 4.5 mpf. **(D)** TUNEL assay indicated increased apoptosis of germ cells in *ar* ^-/-^ testes compared with wildtype (*ar* ^+/+^) siblings at 4.5 mpf. Mpf, months post fertilization. **(E, F)** Expression of *ccnd2a* (*cyclin d2a*) was down-regulated, but expression of *gsdf* was up-regulated in *ar*^-/-^ (*ar ^ihb1225/ihb1225^*) testes compared with wildtype (*ar* ^+/+^) sibling testes (4.5 mpf, n=3, respectively).

Subsequently, we determined expression of germ-cell marker, *vasa* using immunofluorescent staining, which clearly marks germ cells at different stages as indicated by decreased intensity of fluorescence from primordial germ cell (PGC) to mature gametocyte [53]. At 70 dpf, *ar* ^+/+^ testes developed normally as indicated by spermatid (SPD) filling, but, spermatogenesis in *ar* ^−/−^ testes was delayed as confirmed by greater spermatogonia (SG), primary spermatocytes (PSP) and secondary spermatocytes (SSP) (Figure 5A). At 4.5 mpf, many spermatoza (SZ) were filled in *ar* ^+/+^ testes, but *ar* ^−/−^ testes remained filled with spermatogonia (SG), primary spermatocyte (PSP) and secondary spermatocyte (SSP) (Figure 5B). Although it appeared that some spermatozoa (SZ) were present in *ar* ^−/−^ testes, fewer sperm could be isolated from *ar* ^−/−^ males. When homogenous adult *ar* ^−/−^ testes were mixed with eggs from wildtype females, few fertilized eggs were observed (data not shown). These phenotypes were also observed in another *ar* mutant line (M2) (Supplementary Figure 6A). Thus, *ar* ^−/−^ testes are almost dysfunctional.

To determine the causes underlying the effects of *ar* on spermatogenesis, we quantified expression of cell proliferation marker, *cyclin D1* by immunohistochemistry. Figure 5C shows that cell proliferation was greater in *ar* ^+/+^ testes compared with *ar* ^−/−^ testes. In addition, TUNEL assay confirmed more germ cells in *ar* ^−/−^ testes were apoptotic (Figure 5D). Therefore, targeted disruption of *ar* causes progressive loss of spermatogenesis in zebrafish.

To understand how *ar* affects zebrafish spermatogenesis, we determined expression of two genes associated with meiosis. Figure 5E and 5F shows that expression of *ccnd2a* (*cyclin d2a*) was down-regulated, but expression of *gsdf* (*gonadal somatic cell derived factor*), a growth factor expressed in Sertoli cells of testes, was up-regulated in *ar* ^−/−^ (*ar ^ihb1225/ihb1225^*) testes compared with that of wildtype (*ar* ^+/+^) sibling testes (4.5 mpf, n=3, respectively) [54, 55].

### Targeted disruption of *ar* in zebrafish causes premature ovarian failure during growth

**Figure 6:**
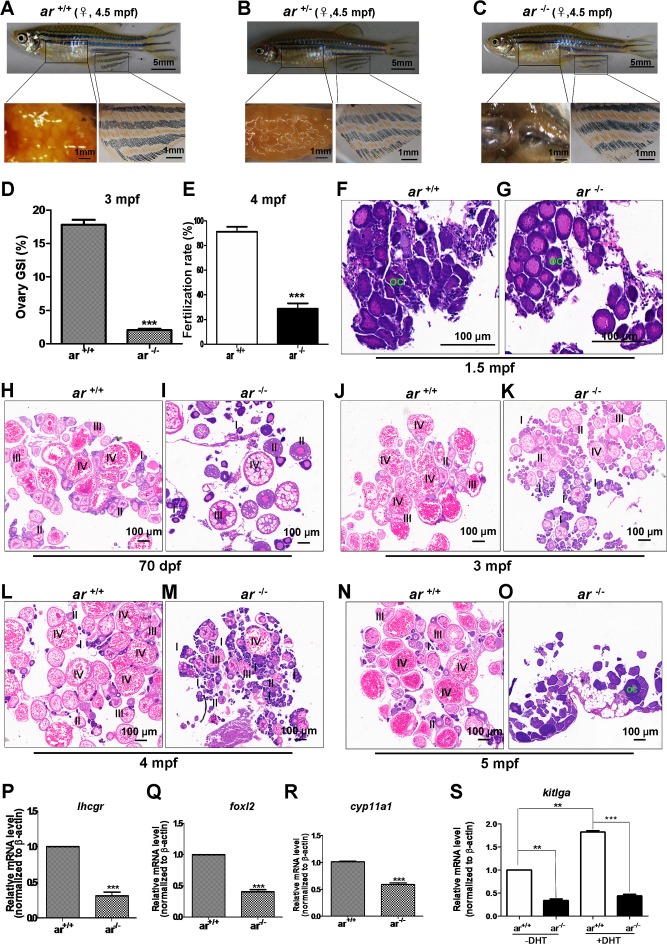
Targeted disruption of *ar* in zebrafish causes premature ovarian failure during growth **(A-C)** Gross appearance and ovaries from wildtype (*ar* ^+/+^), heterozygous (*ar* ^+/-^) and homozygous (*ar* ^-/-^) (*ar ^ihb1225/ihb1225^*) female zebrafish. At 4.5 mpf, the gross appearance of *ar* ^-/-^ (*ar ^ihb1225/ihb1225^*) female zebrafish was indistinguishable from wildtype siblings (*ar* ^+/+^) or their heterozygous sibling (*ar* ^+/-^), but the ovaries of *ar* ^-/-^ female were smaller with fewer eggs and became transparent compared with wildtype sibling (*ar* ^+/+^) or heterozygous siblings (*ar* ^+/-^). **(D)** Gonadosomatic index (GSI) in *ar* ^+/+^ and *ar* ^-/-^ female zebrafish. **(E)** Fertilization rate of eggs from wildtype (*ar* ^+/+^) and homozygous (*ar* ^-/-^) female zebrafish at 4mpf. **(F, G)** Histology of ovaries from wildtype (*ar* ^+/+^) and homozygous (*ar* ^-/-^) (*ar ^ihb1225/ihb1225^*) zebrafish at 1.5 mpf. No obvious difference between *ar* ^-/-^ and *ar* ^+/+^ ovaries. OC, Oocyte. **(H, I)** Histology of ovary from the wildtype (*ar* ^+/+^) and homozygous (*ar* ^-/-^) (*ar ^ihb1225/ihb1225^*) zebrafish at 70 dpf. Ovaries of wildtype (*ar* ^+/+^), most oocytes developed to stage III and IV; but more ovaries of homozygous (*ar* ^-/-^) (M1) developed to stage II or I, some developed to stage III and IV. **(J, K)** Histology of ovaries from wildtype (*ar* ^+/+^) and homozygous (*ar* ^-/-^) zebrafish at 4 mpf. In ovaries of wildtype (*ar* ^+/+^), most oocytes developed to stage IV, but for ovaries of homozygous (*ar* ^-/-^), most oocytes developed to stage II and partial degeneration occurred. **(L, M)** Histology of ovaries from wildtype (*ar*^+/+^) and homozygous (*ar* ^-/-^) zebrafish at 4 mpf. In ovaries of homozygous (*ar* ^-/-^), follicles atresia and degeneration occurred. **(N, O)** Histology of ovaries from wildtype (*ar* ^+/+^) and homozygous (*ar* ^-/-^) zebrafish at 5 mpf. In ovaries of homozygous (*ar* ^-/-^), no follicles were observed and severe degeneration occurred (30%, n = 30). **(P)** *lhcgr* (luteinizing homone/choriogonadotropin receptor) expression in ovaries of wildtype (*ar*^+/+^) and homozygous (*ar*^-/-^) zebrafish at 4.5 mpf. **(Q)** *foxl2* (*forkhead box L2*) expression in ovaries of wildtype (*ar* ^+/+^) and homozygous (*ar* ^-/-^) zebrafish at 4.5 mpf. **(R)** *cyp11a1* (p450 side chain cleavage enzyme) expression in ovaries of wildtype (*ar*^+/+^) and homozygous (*ar* ^-/-^) zebrafish at 4.5 mpf. **(S)** *kitlga* expression in ovaries of wildtype (*ar*^+/+^) and homozygous (*ar* ^-/-^) zebrafish at 4.5 mpf injected with or without DHT (100 nM, 10μl for each). Dpf, days post fertilization; Mpf, months post fertilization.

To understand the effect of *ar* on female reproductive development, histology and immunohistochemistry were used to study ovaries. At 4.5 mpf, gross appearance of *ar* ^−/−^ female was indistinguishable from wildtype (*ar*^+/+^) and heterozygous (*ar* ^+/−^) sibling females (Figure 6A-6C). However, ovaries of *ar*^−/−^ females were smaller with fewer eggs at 4.5 mpf (Figure 6C). Consistently, a significant decrease of the gonadosomatic index (GSI) was found in *ar* ^−/−^ ovaries (Figure 6D). From 3 mpf to 4.5 mpf, the average egg number per female in *ar* ^−/−^ was decreased compared with that of wildtype siblings (Supplementary Figure 4A and 4B). After mating with wildtype males (AB line), less fertilization occurred in *ar* ^−/−^ eggs (Figure 6E). At 1.5 mpf, no obvious difference was noted between wildtype (*ar* ^+/+^) and homozygous (*ar* ^−/−^) (*ar ^ihb1225/ihb1225^*) ovaries (Figure 6F and 6G). At 70 dpf, in wildtype ovaries (*ar*^+/+^), most oocytes developed to stage III and IV; but in ovaries of *ar ^−/−^*, most oocytes developed to stage I or II, and some to III and IV (Figure 6H and 6I). At 3 mpf, in wildtype ovaries (*ar* ^+/+^), most oocytes developed to stage IV; but in ovaries of the homozygous (*ar* ^−/−^), most oocytes developed to stage II and follicles were partially atretic (Figure 6J and 6K). At 4 mpf, in the homozygous (*ar* ^−/−^) ovaries, atretic follicles were observed and degeneration occurred (Figure 6L and 6M). At 3 to 4 mpf, *ar* ^−/−^ ovaries prematurely failed, and most follicles were arrested at stage I or II as indicated by female sub-fertility. In 5 mpf, in *ar ^−/−^* homozygous ovaries (*ar* ^−/−^), no mature oocytes were observed and severe degeneration occurred (30%, n=30). The *ar* ^−/−^ females at this age were completely infertile. These phenotypes were also observed in another *ar* mutant line (*ar ^ihb1226/ihb1226^*) (Supplementary Figure 6B). Thus, targeted disruption of *ar* causes premature ovarian failure.

To understand why disruption of *ar* in zebrafish caused atretic follicles and premature ovarian failure, we determined expression level of folliculogenesis markers, *lhcgr*, *foxl2* and *cyp11a1* in ovaries by qPCR. For 4.5-month old zebrafish ovaries, *lhcgr*, *foxl2* and *cyp11a1* expression was lower in homozygous ovaries (*ar* ^−/−^) (*ar ^ihb1225/ihb1225^*) compared with wildtype siblings (Figure 6P-6R). In addition, *kitlga*, a potential androgen responsive gene and a regulator of folliculogenesis [56–59] was assessed and expression was also lower in homozygous ovaries (*ar* ^−/−^) (*ar ^ihb1225/ihb1225^*) compared with wildtype siblings (Figure 6S). When DHT (100 nM, 10μl each ip) was given, *kitlga* expression was induced significantly in wildtype ovaries (*ar*^+/+^) (Figure 6S). Immunofluorescent assays for ovaries at 4.5 mpf with anti-vasa antibody confirmed histological observations (Supplementary Figure 4C and 4D). Thus, *ar* is essential for the maintenance of zebrafish ovarian function.

### Targeted disruption of *ar* in zebrafish causes reproductive-endocrine disorder

**Figure 7:**
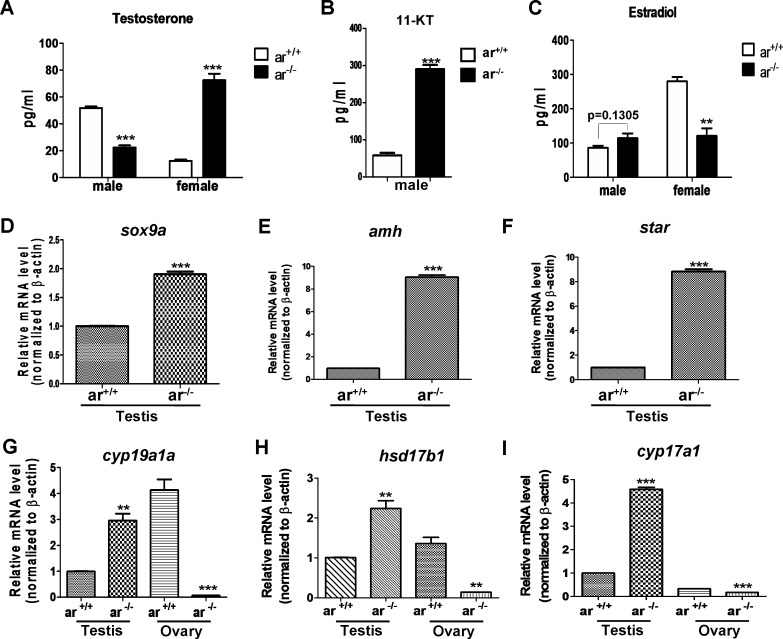
Serum sex hormone and gene expression of key steroidogenic enzymes in wildtype (*ar*^+/+^) and homozygous (*ar*^-/-^) (*ar* ^*ihb1225/ihb1225*^) zebrafish at 5 months (serum hormone) or 4.5 mpf (gene expression) **(A)** In *ar* ^-/-^ male, serum testosterone (T) level was lower, but in *ar* ^-/-^ female, serum testosterone concentration increased. **(B)** In *ar* ^-/-^ male, serum 11-ketotestosterone (11-KT) was higher. **(C)** Serum estradiol (E2) was not altered obviously in *ar* ^+/+^ and *ar* ^-/-^ males, but E2 in *ar* ^+/+^ females is higher than that of *ar* ^-/-^ females. Serum testosterone (T) and estradiol (E2) were measured by ELASA. **(D)** *sox9a* expression in testes from wildtype (*ar* ^+/+^) and homozygous (*ar* ^-/-^) zebrafish. **(E)** *amh* expression in testes from wildtype (*ar* ^+/+^) and homozygous (*ar* ^-/-^) zebrafish. **(F)** *star* expression in testes from wildtype (*ar* ^+/+^) and homozygous (*ar* ^-/-^) zebrafish. **(G)** *cyp19a1a* expression in testes and ovaries from wildtype (*ar* ^+/+^) and homozygous (*ar* ^-/-^) zebrafish. **(H)** *hsd17b1* expression in testes and ovaries from wildtype (*ar* ^+/+^) and homozygous (*ar* ^-/-^) zebrafish. **(I)** *cyp17a1* expression in testes and ovaries from wildtype (*ar* ^+/+^) and homozygous (*ar* ^-/-^) zebrafish. Mpf, months post fertilization.

Given the significant effect of *ar* on zebrafish spermatogenesis and ovarian function, we compared the levels of serum testosterone (T) and estradiol (E2) in mutant and control males and females, respectively. In males (5 mpf), serum testosterone was lower in *ar* ^−/−^ compared with *ar* ^+/+^, but in females (5 mpf), serum testosterone was the opposite for *ar* ^−/−^ and *ar* ^+/+^ (Figure 7A). In addition, in males (5 mpf), serum 11-ketotestosterone (11-KT) was higher in *ar* ^−/−^ compared with *ar* ^+/+^(Figure 7B). Serum estradiol was not different between *ar* ^−/−^ and *ar*^+/+^ males, but was lower in *ar* ^−/−^ females compared with *ar* ^+/+^ females (Figure 7C). Thus, targeted disruption of *ar* in zebrafish causes reproductive-endocrine disorder.

Subsequently, we determined reproductive marker expression *(sox9a* and *amh*) [27, 60, 61], inducers of estradiol (*cyp19a1a* and *hsd17b1*) and hormone synthesis enzymes (*star* and *cyp17a1a*). *sox9a, amh, cyp19a1a*, *hsd17b1*, *star* and *cyp17a1a* expression was higher in *ar* ^−/−^ testes compared with *ar* ^+/+^ testes (Figure 7D-7I). However, in ovaries, *cyp19a1a*, *hsd17b1* and *cyp17a1a* expression was lower in *ar* ^−/−^ compared with *ar*^+/+^ (Figure 7G-7I). Thus, loss of *ar* causes dysregulation of genes related to reproductive development and hormone synthesis.

Therefore, the influence of *ar* KO on zebrafish might arise from disruption of *ar* roles in spermatogenesis, oogenesis and steroid biosynthesis.

## DISCUSSION

The function of *ar in vivo* was first demonstrated in a mouse model [2, 14, 26]. Using tissue-specific male ARKO mice or male global ARKO mice, *ar* was confirmed to be essential for the development of male reproductive organs and spermatogenesis, although not required for testes formation [10, 12]. Female global ARKO mice appear normal, but develop premature ovarian failure (POF) phenotypes, have longer estrous cycles and reduced fertility, fewer follicles, impaired mammary development, delayed production of a first litter and fewer pups per litter [12, 15, 28, 42, 56, 62, 63]. In addition to reproductive defects, ARKO mice have late-onset obesity [14, 44], impaired cardiac growth [64], reduced neutrophils [65], accelerated wound healing [66], reduced bone size, thickness and volume of bone [67], and reduced muscle mass [68].

Even though the zebrafish *ar* gene has been isolated and its expression characterized [45, 46], its function *in vivo* is unclear. Thus, we used CRISPR/Cas9 technology to knock out *ar* in zebrafish and reveal important function *in vivo*. Of note, heterozygous *ar*^+/−^ males were fertile and indistinguishable from wildtype males. In this study, even though we could not verified whether the truncated *ar* proteins were presented in heterozygous *ar*^+/−^ zebrafish or due to lack of suitable zebrafish *ar* antibodies. The same phenotypes between heterozygous and wildtype indicated that knock out of *ar* in zebrafish might cause loss of function of *ar* completely instead of gain of function of the truncated *ar* proteins. In addition, disruption of *ar* resulted in more females; even though this alone does not suggest how zebrafish sex determination occurs, it does confirm the importance of *ar* in zebrafish sex differentiation. Actually, zebrafish sex determination is a complicated affair [47] [49, 69] [70].

It appears that the function of *ar* is evolutionarily conserved because targeted disruption of *ar* in zebrafish caused defects in spermatogenesis and maintenance of ovarian function, which were similar to *ar*-null mice. In addition, late onset of obesity phenotypes as indicated by fatty abdominal cavities were also evident in *ar*-null zebrafish, similar to that of *ar*-null mice [14]. Thus, in zebrafish, androgen receptor might also negatively regulate lipid metabolism. In addition, less energy is spent on gamete production in *ar*-null zebrafish, it might also cause zebrafish to gain weight.

Androgen action on zebrafish organogenesis has been described [71–73] and androgens are required for generation of breeding tubercles (breeding ornaments) on zebrafish pectoral fins [23, 73]. Here, we observed that targeted disruption of *ar* caused defects in the formation of breeding tubercles (BTs), which confirmed the role of androgen action on development of zebrafish secondary sex characteristics. However, we observed no defects of pectoral fin regeneration in *ar*-null male zebrafish, which is reported to be controlled by androgen/GSK3 signaling [71]. Such inconsistency may be due to difference among strains used [33].

Even though the effects of *ar* disruption on reproductive development have been described in a mouse model, the underlying mechanisms are unknown. Once androgens bind to their *ar*, a signaling cascade is initiated that directly or indirectly stimulates reproductive development, but how these genes work and how deregulation of the genes in *ar*-null mice (zebrafish) cause a series of defects is unclear. We noted expression of meiosis-related genes (*ccnd2a* and *gsdf*) were dysregulated in *ar*-null testes and fewer proliferated cells and more apoptotic cells were present. This may partially explain the effect of *ar* on zebrafish spermatogenesis. Also, we noticed that expression of genes involved in reproductive development *(sox9a* and *amh*) [27, 60, 61], estradiol induction (*cyp19a1a* and *hsd17b1*) and hormone synthesis (*star* and *cyp17a1a*) were deregulated in testes and ovaries after *ar* disruption. In addition, in *ar*-null male zebrafish, the level of testosterone was decreased, the level of 11-ketotestosterone was increased. By contrast, in *ar*-null female zebrafish, the level of androgenic sex hormone was increased dramatically, but the level of estrogenic sex hormone was decreased significantly. These phenomena not only suggested that the feminization of *ar*-null zebrafish was mediated by estrogen signaling, but also indicated that the importance of androgen signaling in maintenance of ovary function. The disorder of sexual hormone in *ar*-null zebrafish also confirmed that in zebrafish the synthesis of sexual hormone was inter-conversed during they were synthesized from cholesterol. In *ar*-null zebrafish, when testosterone was reduced, estradiol was increased or *vice versa* [74]. Therefore, dysfunction of the androgen signaling cascade due to *ar* disruption could explain phenotypes seen in *ar*-null zebrafish.

Intriguingly, we noticed some differences between *ar*-null male zebrafish and zebrafish with testis androgen deprivation using trilostane. In zebrafish with testis androgen deprevtion, Leydig cell marked gene (*star*) was increased, but the Sertoli cell gene marked genes (*amh*, *gsdf*) were not changed significantly. It seemed that androgen deprivationmainly affected androgen synthesis in Leydig cells [75]. However, in the testis of *ar*-null zebrafish, 11-KT was increased and the expression of genes related to steroidogenesis was increased in Leydig cells. In addition, the expressions of Sertoli cell marked gene (*amh*), and the spermatogonia-stimulating gene (*gsdf*) were also increased. Given that 11-ketotestosterone (11-KT) instead of testosterone serves as the prominent circulating androgen in zebrafish, the germ cell activities modulated by androgen action might mediate by paracrine factors.

Taken together, *ar*-null zebrafish may be a potential model for revealing pathological mechanisms of diseases related to *ar* defects. Using genetic approaches to understand mechanisms involved in *ar*-modulated reproductive development in a zebrafish model may help to elucidate the role of *ar* in hormone-dependent physiological development and pathogenesis, such as testicular feminization mutation syndrome (Tfm), oligozoospermia and stein-leventhal symdrome.

## MATERIALS AND METHODS

### Zebrafish and their maintenance

The wild type zebrafish (*Danio rerio*; AB strain) were used for generating *ar* mutant lines. The guidelines for zebrafish nomenclature (http://wiki.zfin.org/display/general/ZFIN+Zebrafish+Nomenclature+Guidelines) were followed for naming the two mutants (*ar ^inb1225/ihb1225^* and *ar ^inb1226/ihb1226^*) (https://zfin.org/action/feature/view/ZDB-ALT-161228-1 and https://zfin.org/actin/feature/view/ZDB-ALT-170119-1).

Zebrafish were maintained in a re-circulating water system according to standard protocol. All experiments with zebrafish were approved by the animal care and use committee of Institute of Hydrobiology, Chinese Academy of Sciences.

### Generation of *ar* ^−/−^ zebrafish

The zebrafish-Codon-Optimized Cas9 plasmid [63] was digested with XbaI, purified and transcribed using T7 mMessage Machine Kit (Ambion, Austin, USA). gRNA was designed using http://crispr.mit.edu, and the Exon 4 of *ar* was chosen as a targeting region. pUC19-gRNA scaffold was used for amplifying sgRNA template [76]. The gene name and the primers for PCR amplification were listed in Supplementary Tables 1 and 2. sgRNA was synthesized using Transcript Aid T7 High Yield Transcription Kit (Fermentas, Maryland, USA). Cas9 mRNA and sgRNA were mixed and injected into embryos at one or two-cell stage (500-800ng/μl and 50-80ng/μl, respectively). The mutations were initially detected by HMA (heteroduplex mobility assay) as previously described [77]. If the results were positive, the remainder embryos were raised up to adulthood as the F0, which were back-crossed with wild-type zebrafish (AB line) to generate the F1, which were genotyped by HMA initially and confirmed by sequencing of targeting sites. The heterozygous F1 were back-crossed to wild-type zebrafish (AB line; none of their own parents) to obtain F2. The F2 adult zebrafish carrying the same mutation were inter-crossed to generate the F3 offspring, which should contain wildtype (*ar* ^+/+^), heterozygous (*ar* ^+/−^) and homozygous (*ar*^−/−^).

### Fertility assessment

Egg production assessment was conducted as described previously [78]. To evaluate female fertility between homozygous *ar* ^−/−^ and wildtype (*ar* ^+/+^) siblings, we set 5 groups for *ar* ^−/−^ or *ar*^+/+^ female respectively. One *ar* ^−/−^ or *ar*^+/+^sibling female was mated with two wildtype males (AB line) every day for 2 weeks. The egg numbers were counted and the survival rate was calculated.

For artificial insemination, at 3 mpf, the sperm was obtained by crushing the dissected testes. Briefly, the testes were dissected from mutant male zebrafish under a stereo microscope and transferred to a culture dish. The testes were cut into small pieces in 100 μl of Hanks’ balanced salt solution (0.137 M NaCl, 5.4 mM KCl, 1.3 mM CaCl_2_, 1.0 mM MgSO_4_, 0.25 mM Na_2_HPO_4_, 0.44 mM KH_2_PO_4_, 4.2 mM NaHCO_3_, and 5.55 mM glucose, pH 7.2) and then were crushed using a forceps. Simultaneously, the eggs were obtained from wildtype female zebrafish (AB line). Artificial insemination was carried out according to the method reported previously [79].

For sperm motility evaluation, 1 μl of sperm suspension was placed on a glass slide, then, 1 μl of distilled water was added to the sperm suspension for activating sperms. The sperm motility was observed and evaluated under a dark-phase microscope (Optiphot 2, Nikon Inc., NY, USA) at 40×magnification. The motility value was presented as the percentage of sperms with active movement, the sperms only with vibration were not counted as motile sperms. For each sample, the sperm motility was measured in three different fields at least 2 times.

### Sex determination

For sex determination of the wildtype (*ar* ^+/+^) and homozygous (*ar* ^−/−^) zebrafish, we firstly distinguished them by morphology. Female zebrafish had a big abdomen and prominent genital pore. To ensure the accuracy, we eventually verified their sex by dissection.

### Morphological and histological analysis

Zebrafish (4.5 mpf) were weighted, photographed and dissected after anesthetization. Intact testes and ovaries from zebrafish (1.5 to 5 mpf) were dissected and fixed in 4 % PFA (Paraformaldehyde) over night at 4°C. Samples were dehydrated and embedded in paraffin, and cut into 4 μm. Hematoxylin-Eosin (H.E) staining was performed as described previously [78, 80]. Toluidine blue staining was performed as described previously [81].

### Hormone measurement

Blood samples were collected from 5 month-old zebrafish as described [82]. For each zebrafish, 5 to 10 μl of blood could be collected. Blood collected from 3 individuals was used as one sample for measurement. The blood samples were centrifuged at 5000 g for 20 minutes at 4°C, and the supernatants were separated and purified according to the manufacturer's extraction protocol (Cayman Chemical, Ann Arbor, USA). Testosterone, 11-ketotestosterone and estradiol were measured by competitive enzyme-linked immunosorbent assay (ELISA) kits (Cayman Chemical) following the manufacturer's instructions. All standards and samples were measured by three independent experiments performed in triplicate.

### Immunofluorescent, immunohistochemistry and apoptosis assay

Immunofluorescent staining for germ cells was performed using anti-Vasa antibody (DEAD-box helicase 4 (Cag_Vasa) antibody; Cat# Cag_Vasa, RRID:AB_2631966) as described previously [83, 84]. Immunohistochemistry staining for cell cycle was performed using anti-cyclinD1 antibody (GenWay Biotech Inc. Cat# 18-783-77526, RRID:AB_1009478).

Apoptotic cells were detected by terminal deoxynucleotidyltransferase-mediated dUTP nick end labeling (TUNEL) assay (Roche Applied Science, IN, USA).

### DHT injection assay

Dihydrotestosterone (DHT) (Sigma-Aldrich) were prepared in the vehicle dimethoxyethane (DME) (Sigma-Aldrich, USA) and diluted in Sodium Chloride solution at 100 nM concentration[64]. Three female *ar* ^+/+^ and *ar* ^−/−^ zebrafish (5 months) with similar weight were injected with 10 μl each (100nm/L). After 12 hours of intraperitoneal injection, intact ovaries from adult zebrafish were dissected after anesthetization, and total RNA was extracted from the ovaries for semi-quantitative RT-PCR assays.

### Quantitative real-time PCR analysis

Total RNA was extracted using RNAiso Plus (TaKaRa, Tokyo, Japan) following the protocol provided by the manufacturer. cDNAs were synthesized using the Revert Aid First Strand cDNA Synthesis Kit (Fermentas). SYBR Green mix (Roche) was used for quantitative RT-PCR assays. The gene names and the primers are listed in Supplementary Tables 1 and 2. *Actb1* (*β-actin*) was used as an internal control [85, 86].

### Statistical analysis

Statistical analysis for sex ratio was performed using Microsoft Excel 2007, other statistical analysis was performed using GraphPad Prism, v5 (unpaired *t*-test) (GraphPad Software Inc). Data are reported as means ± S.E.M. of three independent experiments performed in triplicate. The difference was considered to be significant if the p values were less than 0.05. The p values are summarized with the following symbols: ^*^ p<0.05; ^**^ p<0.01; ^***^p<0.001.

## SUPPLEMENTARY MATERIALS FIGURES AND TABLES






