# Supplementary material for: Zebrafish androgen receptor is required for spermatogenesis and maintenance of ovarian function
Source: Oncotarget. 2018 Feb 6;9(36):24320–34. doi: 10.18632/oncotarget.24407 (PMC5966271; doi:10.18632/oncotarget.24407)
Supplement: Supplementary file 1 [file oncotarget-09-24320-s001.pdf]

Zebrafish androgen receptor is required for spermatogenesis and maintenance of ovarian function

SUPPLEMENTARY MATERIALS

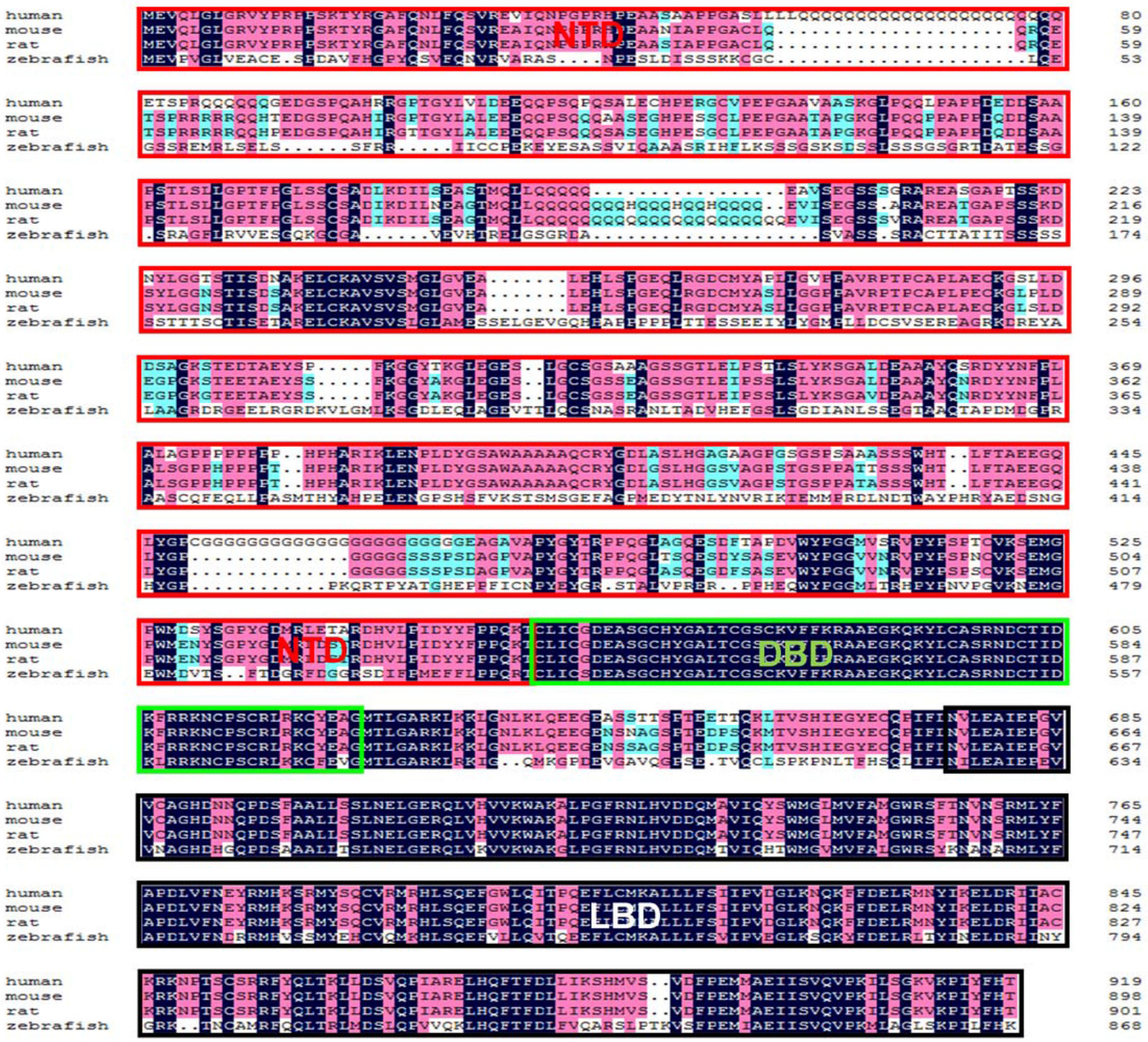

Supplementary Figure 1: Alignment of amino acid sequences of human *AR* (ENSP00000363822), mouse *Ar* (ENSMUSP00000052648), Rat *Ar* (ENSRNOP00000009129) and zebrafish *ar* (ENSDARP00000088795). N-terminal transactivation domain (NTD) is circled by red box; DNA-binding domain (DBD) is circled by green box; ligand-binding domain (LBD) is circled by black box. The same amino acids are marked by deep-blue background.

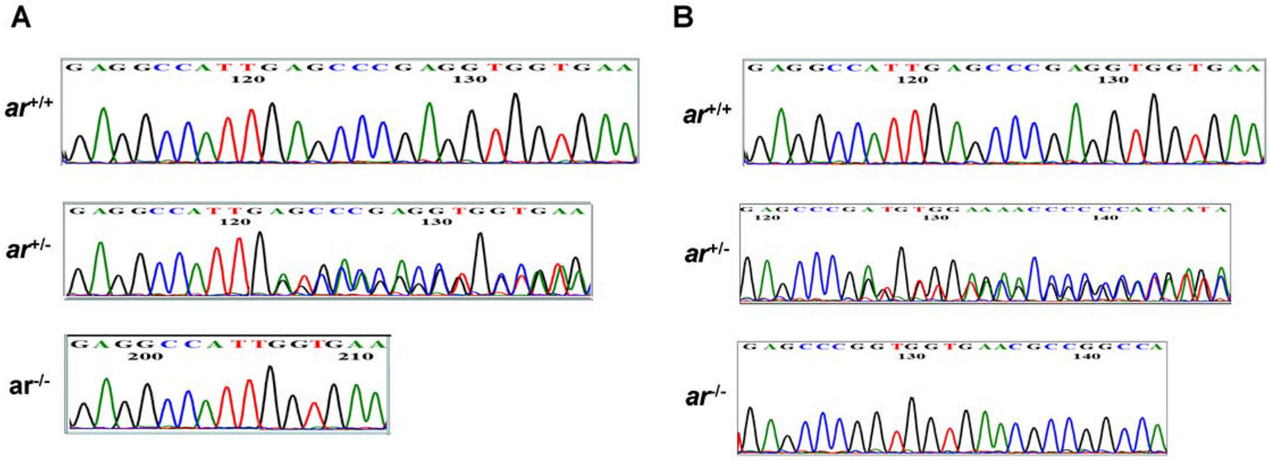

Supplementary Figure 2: Sequencing results of the targeted region from wildtype (*ar* $^{+/+}$), heterozygous (*ar* $^{+/-}$) and homozygous (*ar* $^{-/-}$) zebrafish. (A) The mutant *ar* $^{ihb1225/ihb1225}$. **(B)** The mutant. ar $^{ihb1226/ihb12}$.

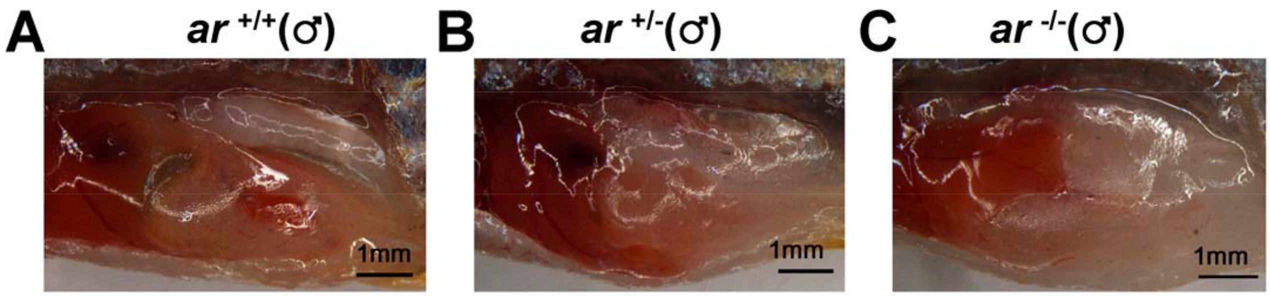

Supplementary Figure 3: General appearance of testis from wildtype (ar $^{+/+}$**), heterozygous (ar** $^{+/-}$**) and homozygous (ar** $^{-/-}$**) zebrafish at 4.5 mpf. (A)** *ar* $^{+/+}$. **(B)** *ar* $^{+/-}$. **(C)** *ar* $^{-/-}$. Mpf, months post fertilization.

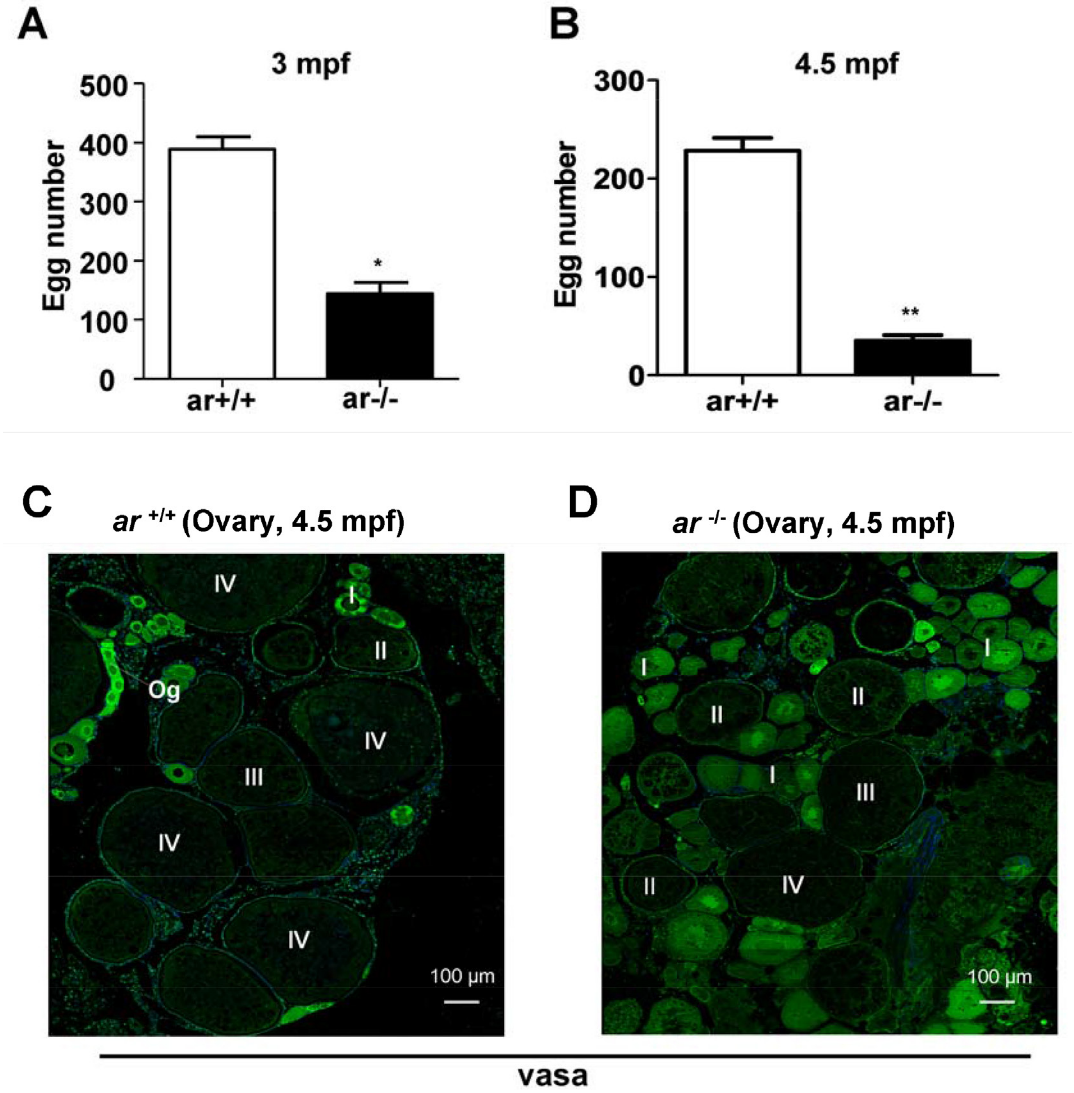

Supplementary Figure 4: (A, B) At 3 or 4.5 mpf, ovary eggs were fewer in *ar* $^{-/-}$ females compared to wildtype siblings (*ar* $^{+/+}$) (n=5 groups, which were measured everyday for 2 weeks). **(C, D)** Immunofluorescent staining using anti-vasa antibody identified different stages of oogenesis in ovaries from wildtype (*ar* $^{+/+}$) and homozygous (*ar* $^{-/-}$) (*ar* $^{ihb1225/ihb1225}$) zebrafish at 4.5 mpf. I, stage I oocyte; II, stage II; III, stage III; IV, stage IV. Mpf, months post fertilization.

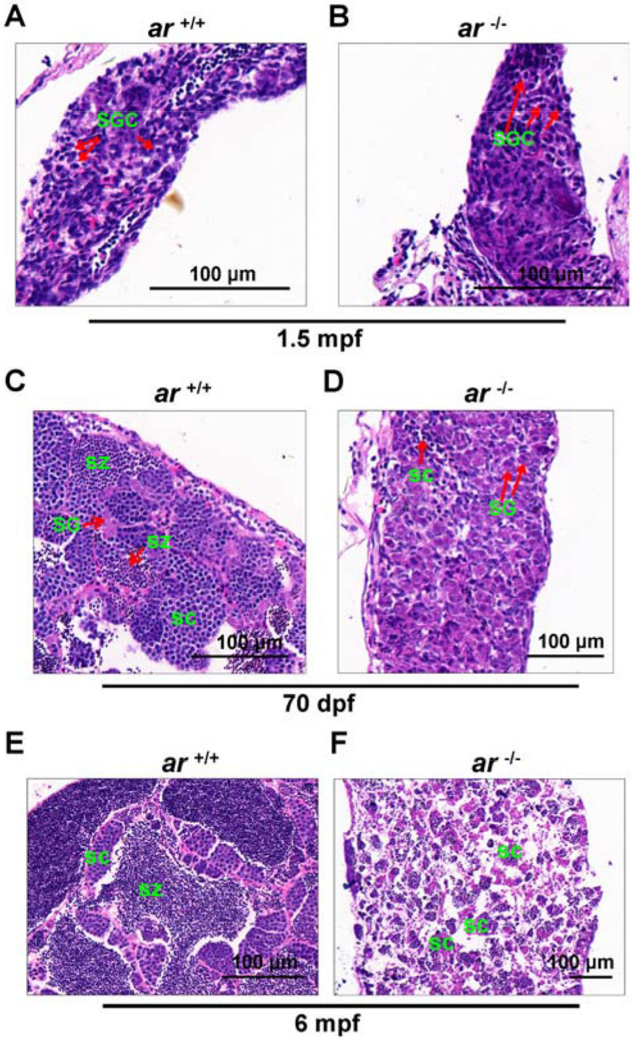

Supplementary Figure 5: Histology of testis from wildtype (*ar* $^{+/+}$), and homozygous (*ar* $^{-/-}$) (the mutant 1) zebrafish at 1.5 mpf, 70dpf and 5 mpf. (A, B) Histology of testes from the wildtype (*ar* $^{+/+}$) and homozygous (*ar* $^{-/-}$) (*ar* $^{ihb1225/ihb1225}$) zebrafish at 1.5 mpf. No obvious difference between *ar* $^{-/-}$ and wildtype sibling (*ar* $^{+/+}$) testes. SGC, spermatogonial cysts (indicated with red arrows). **(C, D)** Histology of testes from the wildtype (*ar* $^{+/+}$) and homozygous (*ar* $^{-/-}$) (*ar* $^{ihb1225/ihb1225}$) zebrafish at 70 dpf. Compared with wildtype sibling testes, spermatogenesis of *ar*$^{-/-}$ testes was delayed as indicated by more spermatogonia (SG) and fewer spermatocytes (SC), but no spermatozoa (SZ). **(E, F)** Histology of testes from the wildtype (*ar* $^{+/+}$) and homozygous (*ar* $^{-/-}$) zebrafish at 6 mpf. *ar* $^{-/-}$ testes were degenerated and loose; but spermatozoa were filled in wildtype sibling (*ar* $^{+/+}$) testes. Dpf, days post fertilization; Mpf, months post fertilization.

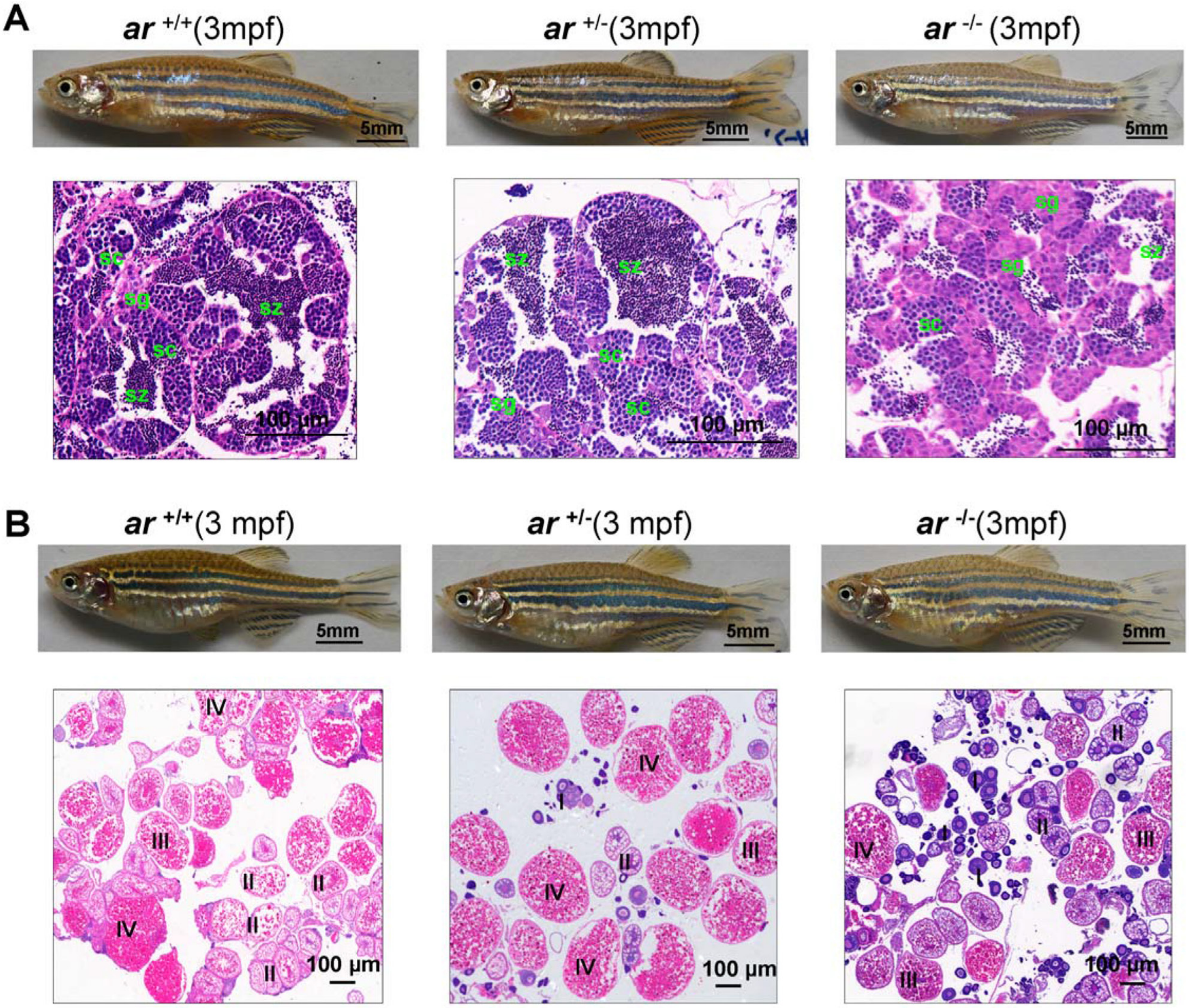

Supplementary Figure 6: Histology of testis and ovary from wildtype (*ar* $^{+/+}$), heterozygous (*ar* $^{+/-}$) and homozygous (*ar* $^{-/-}$) (the mutant 2) zebrafish at 3 mpf. **(A)** Testis. **(B)** Ovary. Mpf, months post fertilization.

Supplementary Table 1: List of gene symbol and gene name used in the text and figures.

See Supplementary File 1

Supplementary Table 2: The primer sequences.

See Supplementary File 2
