# Supplementary material for: Zebrafish androgen receptor is required for spermatogenesis and maintenance of ovarian function
Source: Oncotarget. 2018 Feb 6;9(36):24320–34. doi: 10.18632/oncotarget.24407 (PMC5966271; doi:10.18632/oncotarget.24407)
Supplement: Supplementary file 2 [file oncotarget-09-24320-s002.docx]

| **Gene Symbol** | **Gene name/description** |
| --- | --- |
| *ar* | *androgen receptor* |
| *actb1*(*β-actin*) | *actin, beta 1* |
| *amh* | *anti-Mullerian hormone* |
| *ccnd2a* | *cyclin D2, a* |
| *cyp11a1* | *cytochrome P450, family 11, subfamily A, polypeptide 1* |
| *cyp17a1* | *cytochrome P450, family 17, subfamily A, polypeptide 1* |
| *cyp19a1a* | *cytochrome P450, family 19, subfamily A, polypeptide 1a* |
| *foxl2* | *forkhead box L2* |
| *gsdf* | *gonadal somatic cell derived factor* |
| *hsd17b1* | *hydroxysteroid (17-beta) dehydrogenase 1* |
| *kitlga* | *kit ligand a* |
| *lhcgr* | *luteinizing hormone/choriogonadotropin receptor* |
| *sox9a* | *SRY-box containing gene 9a* |
| *star* | *steroidogenic acute regulatory protein* |

**Supplementary Table 1: List of gene symbol and gene name used in the text and figures**
