# Supplementary material for: Zebrafish androgen receptor is required for spermatogenesis and maintenance of ovarian function
Source: Oncotarget. 2018 Feb 6;9(36):24320–34. doi: 10.18632/oncotarget.24407 (PMC5966271; doi:10.18632/oncotarget.24407)
Supplement: Supplementary file 3 [file oncotarget-09-24320-s003.docx]

**Supplementary Table 2:** **The primer sequences**

| **Primer** | **Sequence (5'-3')** |
| --- | --- |
| gRNA | GTAATACGACTCACTATAGGAGGCCATTGAGCCCGAGGTGGGTTTTAGAGCTAGAAATAGC |
| ar | AAGCAATACTTCCAGCTCTG |
|  | GGCAGGCTTTAATTACCTGG |
| ar-RT | AGTGAAATGGGCCAAAGGAC |
|  | ATCATTGAAGACCAGGTCTGG |
| actb1-RT | TACAATGAGCTCCGTGTTGC |
|  | ACATACAATGGCAGGGGTGTT |
| amh-RT | CTCCTGTTCAGTGTCAATCCTG |
|  | GGCCTGTTATCATCCATCGATG |
| ccnd2a-RT | CAGCACTAACCACTCACTG |
|  | CTGTTCCTGCTGTTGCTG |
| cyp11a1-RT | CAGAGCAATACTGTCCCTC |
|  | CTTGCTCCTGACCTCAATC |
| cyp17a1-RT | ATGCTCCAGTACAGCCAG |
|  | AGAGCATCCAGGAGATCC |
| cyp19a1a-RT | AGATGTCGAGTTAAAGATCCTGCA |
|  | CGACCGGGTGAAAACGTAGA |
| foxl2-RT | AACAGCATCCGACACAAC |
|  | AACATATCCTCGCATGCAG |
| gsdf-RT | AACTCCAGCTGCTGCAGAC |
|  | GTCCATGTAGACGAACGGC |
| hsd17b1-RT | ACCTGAAGAGGACAGAGCC |
|  | TCCATCGCCTCCAGATAC |
| kitlga-RT | AGAAGTGAGTGGCATGTGC |
|  | AGGTCCAATGTGATACCGC |
| lhcgr-RT | ATAGGCGTCAGCAGCTAC |
|  | CAGGATTACGTACTGCAC |
| sox9a-RT | GGTTCCTTCAACGTTCAGC |
|  | GCTGAAGGTGGAGTACAGC |
| star-RT | GGAATGCCTGAGCAGAAG |
|  | ATGCCATTCTGTCCCTGAG |
